# Modelling Amperometric Biosensors Based on Chemically Modified Electrodes

**DOI:** 10.3390/s8084800

**Published:** 2008-08-19

**Authors:** Romas Baronas, Juozas Kulys

**Affiliations:** 1 Department of Software Engineering, Vilnius University, Naugarduko 24, LT-03225 Vilnius, Lithuania; E-mail: romas.baronas@mif.vu.lt; 2 Institute of Mathematics and Informatics, Akademijos 4, LT-08663 Vilnius, Lithuania; 3 Department of Chemistry and Bioengineering, Vilnius Gediminas Technical University, Sauletekio al. 11, LT-10223 Vilnius, Lithuania; E-mail: juozas.kulys@fm.vgtu.lt

**Keywords:** chemically modified electrode, biosensor, modelling, simulation

## Abstract

The response of an amperometric biosensor based on a chemically modified electrode was modelled numerically. A mathematical model of the biosensor is based on a system of non-linear reaction-diffusion equations. The modelling biosensor comprises two compartments: an enzyme layer and an outer diffusion layer. In order to define the main governing parameters the corresponding dimensionless mathematical model was derived. The digital simulation was carried out using the finite difference technique. The adequacy of the model was evaluated using analytical solutions known for very specific cases of the model parameters. By changing model parameters the output results were numerically analyzed at transition and steady state conditions. The influence of the substrate and mediator concentrations as well as of the thicknesses of the enzyme and diffusion layers on the biosensor response was investigated. Calculations showed complex kinetics of the biosensor response, especially when the biosensor acts under a mixed limitation of the diffusion and the enzyme interaction with the substrate.

## 1. Introduction

Biosensors are analytical devices converting a biochemical recognition reaction into a measurable effect [1, 2]. Amperometric biosensors measure the changes in the output current on the working electrode due to the direct oxidation or reduction of products of a biochemical reaction [3]. They, being compact and having relatively short response time, are widely applied to monitor chemical substances in the medicine, food technology and the environmental industry [4, 5].

A large part of commercially available and disposable biosensors is prepared by screen-printing technology [1, 6–8]. They usually contain chemically modified (CM) graphite together with an enzyme [9– 11]. At a CM electrode (CME), electrocatalysis accomplishes by an immobilized redox substance acting as an electron transfer mediator between the graphite electrode and a reaction substrate [12–15].

The understanding of the kinetic peculiarities of biosensors is of crucial importance for their design. The mathematical modelling is rather widely used to improve the efficiency of the biosensors design and to optimize their configuration [16–19]. Starting from seventies various mathematical models of biosensors have been developed and successfully used to study and optimise analytical characteristics of biosensors [20–25]. A comprehensive review on the modelling of amperometric biosensors has been presented by Schulmeister [26]. Mathematical modelling has been also successfully applied for specific sensors based on CME [6, 27–31].

The goal of this investigation was to make a model allowing an effective computer simulation of amperometric biosensors based on CME as well as to investigate the influence of the physical and kinetic parameters on the biosensor response. An ordered ping-pong scheme of the enzyme catalysed substrate conversion in presence of a mediator is considered. The CME is considered as an electrode containing a relatively thin layer of the low soluble mediator and covered with an enzyme membrane. The developed model is based on non-stationary reaction-diffusion equations [32, 33]. It involves three regions: the enzyme layer where an enzymatic reaction as well as the mass transport by diffusion take place, a diffusion limiting region where only the mass transport by diffusion takes place and a convective region where the analyte concentration is maintained constant. In order to define the main governing parameters of the mathematical model the corresponding dimensionless model was derived. By changing input parameters the output results were numerically analyzed at transition and steady state conditions.

## 2. Mathematical Model

We consider an ordered ping-pong scheme of enzyme (E) catalysed substrate (S) conversion in presence of mediator (M),

(1) $${\text{E}}_{\text{ox}}+\;s\overset{k_{1}}{\underset{k_{-1}}{\text{⇌}}}\text{ES}\overset{k_{2}}{\to }{\text{E}}_{\text{red}}+{\text{P}}_{1},$$

(2) $${\text{E}}_{\text{red}}+\;\text{M}\overset{k_{3}}{\to }{\text{E}}_{\text{ox}}+\;\text{P},$$

where E_ox_, E_red_ and ES are oxidized enzyme, reduced enzyme and enzyme substrate, respectively, P and P_1_ are the reaction products.

The reaction takes part on a chemically modified electrode (CME). The CME is considered as an electrode containing a relatively thin layer of the low soluble mediator and covered with an enzyme membrane. The model involves three regions: the enzyme layer where the enzymatic reaction as well as the mass transport by diffusion takes place, a diffusion limiting region where only the mass transport by diffusion takes place and a convective region where the analyte concentration is maintained constant.

### 2.1. Governing Equations

Assuming the quasi steady state approximation, the concentration of the intermediate complex (ES) do not change and is usually neglected when simulating the biochemical behaviour of biosensors [1, 2]. Additionally assuming the symmetrical geometry of the electrode and homogeneous distribution of the immobilized enzyme in the enzyme layer of uniform thickness, the mass transport and the kinetics in the enzyme layer can be expressed by a system of reaction-diffusion equations (*t* > 0),

(3) $$\frac{\partial s_{e}}{\partial t}=D_{se}\frac{\partial^{2}s_{e}}{\partial x^{2}}-\upsilon \left(m_{e},s_{e}\right),$$

(4) $$\frac{\partial m_{e}}{\partial t}=D_{\text{me}}\frac{\partial^{2}m_{e}}{\partial x^{2}}-\upsilon \left(m_{e},s_{e}\right),$$

(5) $$\frac{\partial p_{e}}{\partial t}=D_{pe}\frac{\partial^{2}p_{e}}{\partial x^{2}}+\upsilon \left(m_{e},s_{e}\right),\;0<x<d_{e},$$

where *x* stands for space, *t* stands for time, *s_e_*(*x*,*t*), *m_e_*(*x*,*t*), *p_e_*(*x*,*t*) are the concentrations of the substrate, mediator and reaction product, respectively, *d_e_* is thickness of the enzyme layer, *D_se_*, *D_me_* and *D_pe_* are the diffusion coefficients for the substrate, mediator and reaction product, respectively, and *υ*(*m_e_*, *s_e_*) is the quasi steady state enzyme reaction rate for the ordered ping-pong scheme (1) and (2). According to the scheme:

(6) $$\frac{e_{t}}{\upsilon }=\frac{1}{k_{\text{cat}}}+\frac{1}{k_{\text{red}}s_{e}}+\frac{1}{k_{\text{ox}}m_{e}},$$

where *e_t_* is the total concentration of enzyme, *k_cat_* is catalytic constant of ES conversion, *k_cat_*= *k*_2_, *k_red_* is an apparent bimolecular constant of the enzyme and substrate interaction, *k_red_*= *k*_1_*k*_2_/(*k*_−1_ + *k*_2_), *k_ox_* is a constant of the enzyme interaction with the mediator, *k_ox_*= *k*_3_. The total sum *e_t_* of the concentrations of all the enzyme forms is assumed to be constant in the entire enzyme layer, *e_t_* = *e_ox_* + *e_red_* + *e_s_*, where *e_ox_*, *e_red_*, *e_s_* are the concentrations of E_ox_, E_red_, ES, respectively. From (6) we obtain the following non-linear expression of the reaction rate:

(7) $$\upsilon \left(m_{e},s_{e}\right)=\frac{e_{t}k_{\text{cat}}k_{\text{red}}k_{ox}m_{e}s_{e}}{k_{\text{red}}k_{\text{ox}}m_{e}s_{e}+k_{\text{cat}}k_{\text{ox}}m_{e}+k_{\text{cat}}k_{\text{red}}s_{e}}.$$

Outside the enzyme layer only the mass transport by diffusion takes place. We assume that the outer mass transport obeys a finite diffusion regime,

(8) $$\frac{\partial s_{d}}{\partial t}=D_{\text{sd}}\frac{\partial^{2}s_{d}}{\partial x^{2}},$$

(9) $$\frac{\partial m_{d}}{\partial t}=D_{\text{md}}\frac{\partial^{2}m_{d}}{\partial x^{2}},$$

(10) $$\frac{\partial p_{d}}{\partial t}=D_{\text{pd}}\frac{\partial^{2}p_{d}}{\partial x^{2}},\;d_{e}<x<d_{e}+d_{d},$$

where *s_d_*(*x*, *t*), *m_d_*(*x*, *t*) and *p_d_*(*x*, *t*) are the concentrations of the substrate, mediator and product, respectively, in the diffusion layer, *d_d_* is the thickness of the diffusion layer, *D_sd_*, *D_md_*, *D_pd_* are the diffusion coefficients.

The diffusion layer (*d_e_*< *x* < *d_e_*+*d_d_*) may be treated as the Nernst diffusion layer [32, 33]. According to the Nernst approach a layer of thickness *d_d_* remains unchanged with time. Away from it the solution is in motion and uniform in concentration.

### 2.2. Initial Conditions

Let *x* = 0 represents the surface of the CME, while *x* = *d_e_* - the boundary between the enzyme membrane and the buffer solution. The biosenor operation starts when some substrate appears in the bulk solution. This is used in the initial conditions (*t* = 0),

(11) $$s_{e}\left(x,0\right)=0,\;0\leq x\leq d_{e},$$

(12) $$m_{e}\left(x,0\right)=\{\begin{array}{c} m_{0},\;x=0,\\ 0,\;0<x\leq d_{e}, \end{array}$$

(13) $$p_{e}\left(x,0\right)=0,\;0\leq x\leq d_{e},$$

(14) $$s_{d}\left(x,0\right)=\{\begin{array}{c} 0,\;d_{e}\leq x<d_{e}+d_{d},\\ s_{0},\;x=d_{e}+d_{d}, \end{array}$$

(15) $$m_{d}\left(x,0\right)=0,\;d_{e}\leq x\leq d_{e}+d_{d},$$

(16) $$p_{d}\left(x,0\right)=0,\;d_{e}\leq x\leq d_{e}+d_{d},$$

where *m*_0_ is the concentration of the mediator at the boundary between electrode and enzyme layer, *s*_0_ is the concentration of the substrate in the bulk solution.

### 2.3. Boundary Conditions

On the boundary between two regions having different diffusivities, we define the matching conditions (*t* > 0),

(17) $${D_{se}\frac{\partial s_{e}}{\partial x}|}_{x=d_{e}}={D_{sd}\frac{\partial s_{d}}{\partial x}|}_{x=d_{e}},\;s_{e}\left(d_{e},t\right)=s_{d}\left(d_{e},t\right),$$

(18) $${\;D_{me}\frac{\partial m_{e}}{\partial x}|}_{x=d_{e}}{=D_{md}\frac{\partial m_{d}}{\partial x}|}_{x=d_{e}},\;m_{e}\left(d_{e},t\right)=m_{d}\left(d_{e},t\right),$$

(19) $${D_{pe}\frac{\partial p_{e}}{\partial x}|}_{x=d_{e}}{=D_{pd}\frac{\partial p_{d}}{\partial x}|}_{x=d_{e}},\;p_{e}\left(d_{e},t\right)=p_{d}\left(d_{e},t\right).$$

These conditions mean that fluxes of the substrate, mediator and product through the stagnant external layer equal to the corresponding fluxes entering the surface of the enzyme membrane. The partition of the substrate, mediator and product in the membrane versus the bulk is assumed to be equal.

In the bulk solution the concentrations of the substrate, mediator and product remain constant (*t* > 0),

(20) $$s_{d}\left(d_{e}+d_{d},t\right)=s_{0},$$

(21) $$m_{d}\left(d_{e}+d_{d},t\right)=0,$$

(22) $$p_{d}\left(d_{e}+d_{d},t\right)=0.$$

The concentration *p_e_* of the reaction product at the electrode surface (*x* = 0) is being permanently reduced to zero due to the electrode polarization. Following the scheme (1), (2), the substrate is an electro-inactive substance. The concentration of the mediator covering the electrode surface is kept constant. This is described by the following boundary conditions (*t* > 0):

(23) $$D_{se}{\frac{\partial s_{e}}{\partial x}|}_{x=0}=0,$$

(24) $$m_{e}\left(0,t\right)=m_{0},$$

(25) $$p_{e}\left(0,t\right)=0.$$

The constant concentration *m*_0_ of the mediator on the electrode can be achieved by permanent dissolution of adsorbed mediator. The direct measurements show that *m*_0_ can be as low as 10^-6^ M [34].

### 2.4. Biosensor Response

The measured current is accepted as a response of an amperometric biosensor in physical experiments. The anodic current is directly proportional to the flux of the reaction product at the electrode surface [1, 2], i.e. on the border *x* = 0. Since the total current is also directly proportional to the area of the electrode surface we normalize the total current with the area of that surface. The density *i*(*t*) of the biosensor current at time *t* can be obtained explicitly from the Faraday's and Fick's laws,

(26) $$i\left(t\right)=n_{e}{\text{F D}}_{pe}{\frac{\partial_{P_{e}}}{\partial x}|}_{x=0},$$

where *n_e_* is a number of electrons involved in a charge transfer at the electrode surface, and *F* is Faraday constant, *F* = 96485 C/mol. We assume that the system (3)-(5), (8)-(25) approaches a steady state as *t* → ∞,

(27) $$i_{s}=\underset{t\to \infty }{\lim}i\left(t\right),$$

where *i_S_* is assumed as the steady state biosensor current.

The sensitivity is also a very important characteristic of biosensors [1–5]. The biosensor sensitivity is defined as a gradient of the steady state current with respect to the substrate concentration in the bulk solution. The biosensor current as well as the substrate concentration vary in orders of magnitude. Therefore, a dimensionless expression of the sensitivity is preferable,

(28) $$B_{S}\left(s_{0}\right)=\frac{di_{S}\left(s_{0}\right)}{ds_{0}}\times \frac{s_{0}}{i_{S}\left(s_{0}\right)},$$

where *B_S_*(*s*_0_) stands for the dimensionless sensitivity of the biosensor at the concentration *s*_0_ of the substrate in the bulk solution, *i_S_*(*s*_0_) is the steady state current calculated at the substrate concentration *S*_0_.

The concentrations *s*, *m* and *p* of the substrate, of the mediator and of the reaction product, respectively, can be defined in entire domain *x* ϵ [0, *d_e_* + *d_d_*] as follows (*t* ≥ 0) :

(29) $$u(x,t)=\{\begin{array}{l} u_{e}\left(x,t\right),\;x\in \left[0,d_{e}\right],\\ u_{d}\left(x,t\right),\;x\in (d_{e},d_{e}+d_{d}], \end{array}\;u=s,m,p.$$

All the concentration functions (*s*, *m* and *p*) are continuous in the entire domain *x* ϵ [0, *d_e_* + *d_d_*].

## 3. Numerical Solution

Definite problems arise when solving analytically non-linear partial differential equations [32]. Be-cause of this the problem was solved numerically using the finite difference technique [33, 35]. To find a numerical solution of the problem we introduced a non-uniform discrete grid in both directions: *x* and *t*.

A semi-implicit linear finite difference scheme has been built as a result of the difference approxima-tion [36, 37]. The resulting system of linear algebraic equations was solved rather efficiently because of the tridiagonality of the matrix of the system.

To have an accurate and stable result it was required to use very small step size in *x* direction at the boundaries *x* = 0, *x* = *d_e_* and *x* = *d_e_* + *d_d_*. We assumed that farther from all these peculiar boundaries, the step size may increase. An exponentially increasing step size was used form 0 to *d_e_*/2, from *d_e_* + *d_d_* down to *d_e_* + *d_d_*/2, from the boundary *d_e_* to both sides: *d_e_* + *d_d_*/2 and *d_e_*/2.

Usually an implicit computational scheme does not restrict time increment [35]. However, the step size in the direction of time was restricted due to the non-linear reaction term (7) and the boundary conditions. In order to achieve accurate and stable solution of the problem, at the very beginning of the reaction-diffusion process we employed the restriction condition which is usually used for fully explicit schemes. Since the biosensor action obeys the steady-state assumption when *t* → ∞, it was reasonable to apply an increasing step size in the time direction. The final step size in time was in a few orders of magnitude higher than the fist one.

The digital simulator has been programmed in JAVA language [38].

In digital simulation, the biosensor response time was assumed as the time when the absolute current slope value falls below a given small value normalized with the current value. In other words, the time needed to achieve a given dimensionless decay rate *ε* was used,

(30) $$t_{R}{=}_{i\left(t\right)>0}^{\text{min}}\left\{t:\frac{1}{i\left(t\right)}\left|\frac{di\left(t\right)}{dt}\right|<\varepsilon \right\},\;i(t_{R})\approx i_{S,}$$

where *t_R_* is assumed as the response time. In calculations, we used *ε* = 10^-4^. The response time *t_R_* as an approximate steady state time is very sensitive to the decay rate *ε*, i.e. *t_R_* → ∞ when *ε* → 0. Because of this, we employed a half of steady state time to investigate the behavior the response time [32]. The resultant relative output signal function i*(*t*) can be expressed as follows:

(31) $$i^{*}\left(t\right)=\frac{i\left(t_{R}\right)-i\left(t\right)}{i\left(t_{R}\right)},\;0\leq i^{*}\left(t\right)\leq 1,\;t>0.$$

Let *t*_0 5_ be the time at which the reaction-diffusion process reaches the medium, called the half time of the steady state or, particularly, half of the time moment of occurrence of the steady state current, i.e. i*(*t*_0.5_) = 0.5, *i*(*t*_0.5_) = 0.5*i*(*t_R_*).

## 4. Model Validation

The adequacy of the mathematical model of the biosensor was evaluated using known analytical solution of a two compartment model of amperometric biosensors [26]. As one can see from the reaction rate *υ* introduced by (7) that the kinetics of the biochemical reaction significantly depends on the ratio of the substrate and the mediator concentrations. Let us introduce the dimensionless ratio Σ of the substrate (*s*_0_) to the mediator (*m*_0_) concentrations combining them with the rates of the corresponding reactions (1) and (2),

(32) $$\sum =\frac{s_{0}k_{\text{red}}}{m_{0}k_{ox}}$$

At relatively low concentrations of the substrate when Σ ≪ 1 (*s*_0_*k_red_* ≪ *m*_0_*k_ox_*), the reaction rate *υ*(*m*, *s*) introduced by (7) reduces as follows:

(33) $$\upsilon \left(m,s\right)\approx \frac{e_{t}k_{\text{cat}}k_{\text{red}}s}{k_{\text{red}}s+k_{\text{cat}}}.$$

Consequently, in this case the mediator concentration makes no notable effect on the product concentration, and the governing equation (4) can be neglected when simulating the biosensor response.

Assuming (33), the governing equations (3), (5), (8), (10), together with the initial conditions (11), (13), (14), (16) and the boundary conditions (17), (19), (20), (22), (23), (25) form a boundary value problem which can be solved analytically in the cases when the reaction function (33) approaches to a linear function [26]. At so low concentrations of the substrate as *s*_0_ ≪ *k_cat_*/*k_red_* the reaction rate *υ*(*m*, *s*) reduces further to *e_t_k_red_s*. Assuming *υ*(*m*, *s*) ≈ *e_t_k_red_s*, the steady-state current *i_S_* can be calculated as follows [26]:

(34) $$\begin{array}{l} i_{S}=n_{e}FD_{\text{pe}}s_{0}\frac{1}{d_{e}+d_{d}}\left(d_{e}+d_{d}\times \frac{D_{\text{sd}}-\sigma_{\text{red}}D_{\text{se}}\sinh\left(\sigma_{\text{red}}\right)/\cosh\left(\sigma_{\text{red}}\right)}{D_{\text{sd}}+\sigma_{\text{red}}D_{\text{se}}\left(d_{d}/d_{e}\right)\sinh\left(\sigma_{\text{red}}\right)/\cosh\left(\sigma_{\text{red}}\right)}\right)\times \\ \left(\frac{\sigma_{\text{red}}D_{\text{se}}d_{d}}{d_{e}}\times \frac{\sinh\left(\sigma_{\text{red}}\right)}{\cosh\left(\sigma_{\text{red}}\right)}+\frac{D_{\text{se}}D_{\text{pd}}}{D_{\text{pe}}}\left(1-\frac{1}{\cosh(\sigma_{\text{red}}}\right)\right)/\left(D_{\text{pd}}d_{e}+D_{\text{pe}}d_{d}\right), \end{array}$$

(35) $$\sigma_{\text{red}}^{2}=k_{\text{red}}Q,\;Q=\frac{e_{t}d_{e}^{2}}{D_{\text{se}}}.$$

The dimensionless factor $\sigma_{\text{red}}^{2}$ is known as the diffusion modulus or Damköhler number [32]. The diffusion modulus essentially compares the rate (*e_t_k_red_*) of the enzyme reaction with the diffusion rate $(D_{\text{se}}/d_{\text{e}}^{2})$.

The mathematical model as well as the numerical solution of the model was evaluated for different concentrations of the mediator (*m*_0_) and the substrate (*s*_0_). The following values of the model parameters were constant in the numerical simulation:

(36) $$\begin{array}{c} D_{\text{se}}=D_{\text{me}}=D_{\text{pe}}=300\mu {\text{m}}^{2}s^{-1},\;D_{\text{sd}}=2D_{\text{se}},\;D_{\text{md}}=2D_{\text{me}},\;D_{\text{pd}}=2D_{\text{pe}},\\ k_{\text{cat}}=10^{3}s^{-1},\;k_{\text{red}}=10^{4}{\text{M}}^{-1}s^{-1},\;k_{\text{ox}}=10^{7}{\text{M}}^{-1}s^{-1},\;e_{t}=3\mu {\text{M},\;n}_{e}=1. \end{array}$$

The numerical solution of the model (3)-(5), (8)-(25) was compared with the analytical solution (34). At *d_e_* = 100 *μ*m, *d_d_* = 300 *μ*m, *s*_0_ = 1 M and *m*_0_ = 1mM, the relative difference between the numerical and analytical solutions was about 0.5%.

At relatively low concentrations of the mediator when Σ ≫ 1 (*m*_0_*k_ox_* ≪ *s*_0_*k_red_*), the reaction rate *υ*(*m*, *s*) reduces to

(37) $$\upsilon \left(m,s\right)\approx \frac{e_{t}k_{\text{cat}}k_{\text{ox}}s}{k_{\text{ox}}m+k_{\text{cat}}}.$$

In this case the substrate concentration may be neglected when simulating the biosensor response.

Assuming (37), the governing equations (4), (5), (9), (10), together with the initial conditions (12), (13), (15), (16) and the boundary conditions (18), (19), (21), (22), (24), (25) form a boundary value problem which can be solved analytically in the cases when the reaction function (37) approaches to a linear function [6]. At concentrations of the mediator as low as *m*_0_ ≪ *k_cat_*/*k_ox_* and zero thickness of the external diffusion layer, *d_d_* = 0, the steady-state current *i_S_* can be calculated as follows [6]:

(38) $$i_{S}=n_{e}FD_{\text{pe}}m_{0}\frac{1}{d_{e}}\left(\sigma_{\text{ox}}\coth\left(\sigma_{\text{ox}}\right)-1\right),$$

(39) $$\sigma_{\text{ox}}^{2}=k_{\text{ox}}Q.$$

At *d_e_* = 100 *μ*m, *d_d_* = 0, *m*_0_ = 1 *μ*M and *s*_0_ = 0.1 mM, the relative difference between the numerical and analytical solutions was about 1%.

The number *Q* introduced by (35) incorporates the diffusion rate $(D_{\text{se}}/d_{e}^{2})$ and the total concentration *e_t_* of the enzyme. *Q* includes all the parts of the diffusion modulus except the constant *k_red_* of the enzyme - substrate interaction and the constant *k_ox_* of the enzyme-mediator interaction. Assuming constant values of *k_red_* as well as of *k_ox_*, the number *Q* can be used as a reduced diffusion modulus instead of two modulus *σ_red_* and *σ_ox_*.

It is rather well known that an ordinary enzyme electrode acts under diffusion limitation when the diffusion modulus is much greater than unity [26, 39]. If the diffusion modulus is significantly less than unity then the enzyme kinetics predominates in the biosensor response.

In the case of CM electrode, the kinetics of the enzymatic reaction was expressed by two rates: *k_red_* and *k_ox_*. These two rates of the reactions (1) and (2) lead to two diffusion modulus: *σ_red_* and *σ_ox_*. Assuming *k_red_*< *k_ox_* and taking into consideration definitions (35) and (39), we can state that the biosensor acts under limitation of the enzyme-mediator interaction when $Q\ll 1/k_{\text{ox}}\;(\sigma_{\text{ox}}^{2}\ll 1)$. If $Q\gg 1/k_{\text{red}}\;(\sigma_{\text{red}}^{2}\gg 1)$ then the response is under control of the mass transport by diffusion. At intermediate values of *Q* (1/*k_ox_* < *Q* < 1/*k_red_*) the biosensor acts under mixed limitation of the diffusion and the enzyme-substrate interaction.

## 5. Dimensionless Model

In order to define the main governing parameters of the mathematical model we introduce the following dimensionless parameters:

(40) $$X=\frac{x}{d_{e}},\;T=\frac{tD_{\text{se}}}{d_{e}^{2}},\;\delta =\frac{d_{d}}{d_{e}},\;T_{0.5}=\frac{t_{0.5}D_{\text{se}}}{d_{e}^{2}},$$

(41) $$S=\frac{k_{\text{red}}s}{k_{\text{cat}}},\;M=\frac{k_{\text{ox}}m}{k_{\text{cat}}},\;P=\frac{k_{\text{ox}}p}{k_{\text{cat}}},\;S_{0}=\frac{k_{\text{red}}s_{0}}{k_{\text{cat}}},\;M_{0}\frac{k_{\text{ox}}m_{0}}{k_{\text{cat}}},$$

where *s*, *p* and *m* are the concentrations introduced by (29), *X* is the dimensionless distance from the electrode surface, *T* stands for the dimensionless time, *δ* is the dimensionless thickness of the diffusion layer, and *S*, *M*, *P*, *S*_0_, *M*_0_ are the dimensionless concentrations. The dimensionless thickness of enzyme membrane equals one.

The governing equations (3)-(5) in dimensionless coordinates are expressed as follows:

(42) $$\frac{\partial S}{\partial T}=\frac{\partial^{2}S}{\partial X^{2}}-\sigma_{\text{red}}^{2}\frac{\text{MS}}{\text{MS}+M+S},$$

(43) $$\frac{\partial M}{\partial T}=\frac{D_{\text{me}}}{D_{\text{se}}}\frac{\partial^{2}M}{\partial X^{2}}-\sigma_{\text{ox}}^{2}\frac{\text{MS}}{\text{MS}+M+S},$$

(44) $$\frac{\partial P}{\partial T}=\frac{D_{\text{pe}}}{D_{\text{se}}}\frac{\partial^{2}P}{\partial X^{2}}-\sigma_{\text{ox}}^{2}\frac{\text{MS}}{\text{MS}+M+S},\;0<X<1,\;T>0.$$

The governing equations (8)-(10) take the following equations:

(45) $$\frac{\partial S}{\partial T}=\frac{D_{\text{sd}}}{D_{\text{se}}}\frac{\partial^{2}S}{\partial X^{2}},\;\frac{\partial M}{\partial T}=\frac{D_{\text{md}}}{D_{\text{se}}}\frac{\partial^{2}M}{\partial X^{2}},\;\frac{\partial P}{\partial T}=\frac{D_{\text{pd}}}{D_{\text{se}}}\frac{\partial^{2}P}{\partial X^{2}},\;1<X<1+\delta ,\;T>0.$$

The initial conditions (11)-(16) transform to the following conditions:

(46) $$S\left(X,0\right)=\{\begin{array}{c} 0,\;0\leq x<1+\delta ,\\ S_{0},\;X=1+\delta , \end{array}$$

(47) $$M\left(X,0\right)=\{\begin{array}{c} M_{0},\;X=0,\\ 0,\;0<X\leq 1+\delta , \end{array}$$

(48) P(X,0)=0,1≤X≤1+δ,

The matching (17)-(19) and the boundary (20)-(25) conditions are rewritten as follows (*T* > 0):

(49) $${\frac{\partial S}{\partial X}|}_{X=1^{-}}={\frac{D_{\text{sd}}}{D_{\text{se}}}\frac{\partial S}{\partial X}|}_{x=1^{+}}{,\;\frac{\partial M}{\partial X}|}_{X=1^{-}}{=\frac{D_{\text{md}}}{D_{\text{me}}}\frac{\partial M}{\partial X}|}_{x=1^{+}}{,\;\frac{\partial P}{\partial X}|}_{X=1^{-}}={\frac{D_{\text{pd}}}{D_{\text{pe}}}\frac{\partial P}{\partial X}|}_{x=1^{+}},$$

(50) $${\frac{\partial S}{\partial X}|}_{X=0}=0,\;M\left(0,T\right)=M_{0,\;}P\left(0,T\right)=0,$$

(51) $$S\left(1+\delta ,T\right)=S_{0,\;}M\left(1+\delta ,T\right)=0,\;P\left(1+\delta ,T\right)=0.$$

The dimensionless current (flux) *I* and the corresponding dimensionless stationary current *I_S_* are defined as follows:

(52) $$I\left(T\right)={\frac{\partial P}{\partial X}|}_{X=0}=\frac{i\left(t\right)k_{\text{ox}}d_{e}}{n_{e}FD_{\text{pe}}k_{\text{cat}}},\;I_{S}=\lim_{T\to \infty }I\left(T\right),$$

Assuming the same diffusion coefficients for the all three species, only the following dimensionless parameters remain in the dimensionless mathematical model (42)-(51): *δ* - the thickness of the diffusion layer, *S*_0_ - the substrate concentration in the bulk solution, *M*_0_ - the mediator concentration at the elec-trode surface, *σ_ox_* and *σ_red_* - the diffusion modulus, and *D_rel_* - the ratio of the external diffusivity to the internal diffusivity, *D_rel_* = *D_sd_*/*D_se_* = *D_md_*/*D_me_* = *D_pd_*/*D_pe_*. In all the calculations we used *D_rel_* = 2 as defined by (36). As it was mentioned above, it is reasonable to use the reduced diffusion modulus *Q* instead of two modulus: *σ_ox_* and *σ_red_*.

## 6. Results and Discussion

Using numerical simulation, peculiarities of the biosensor action has been investigated at different values of the model parameters.

### 6.1. The Dynamics of the Biosensor Action

**Figure 1.**
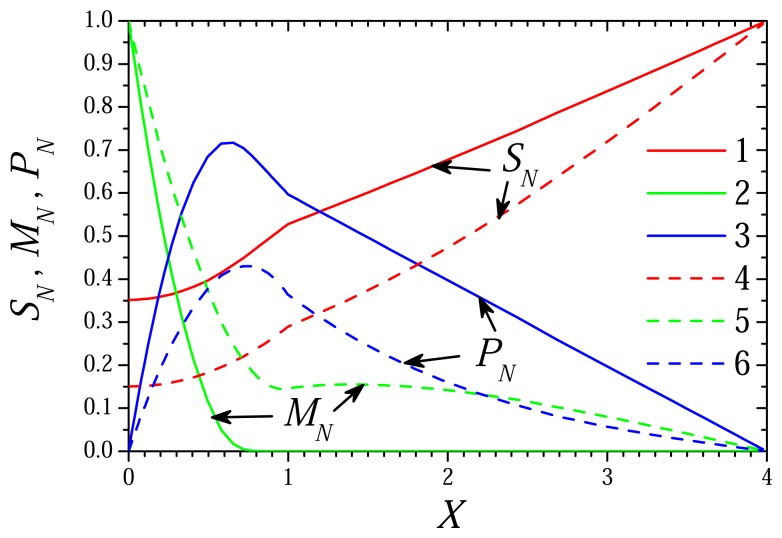
Profiles of the normalized concentrations of the substrate (1, 4), mediator (2, 5) and product (3, 6) in the enzyme layer *X* ∈ (0,1) and in the diffusion layer *X* ∈ (1,4) at approximate steady state dimensionless time *T_R_* = 5.73 (1-3) and the dimensionless half-time *T*_0.5_ = 1.86 (4-6), *S*_0_= 0.1, *M*_0_ = 10.

**Figure 2.**
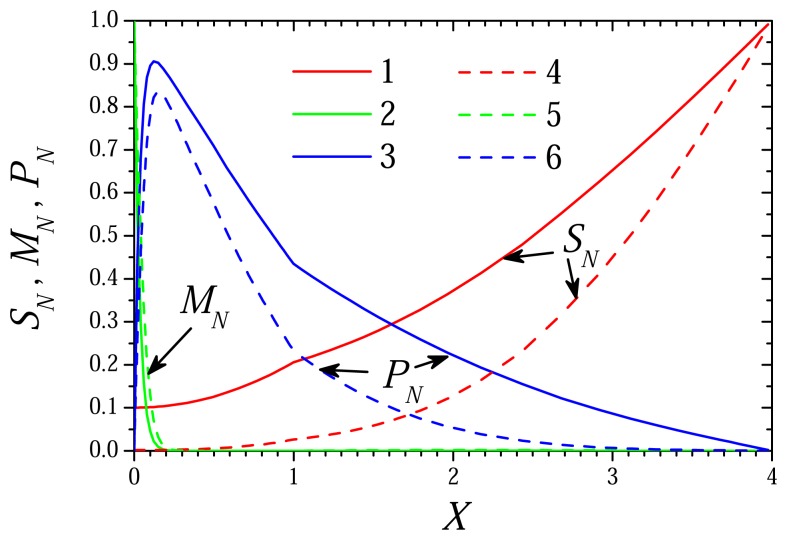
Profiles of the normalized concentrations of the substrate (1, 4), mediator (2, 5) and product (3, 6) in the enzyme and diffusion layers at approximate steady state dimensionless time *T_R_* = 1.2 (1-3) and the dimensionless half-time *T*_0.5_ = 0.435 (4-6), *S*_0_ = 10, *M*_0_ = 0.1.

**Figure 3.**
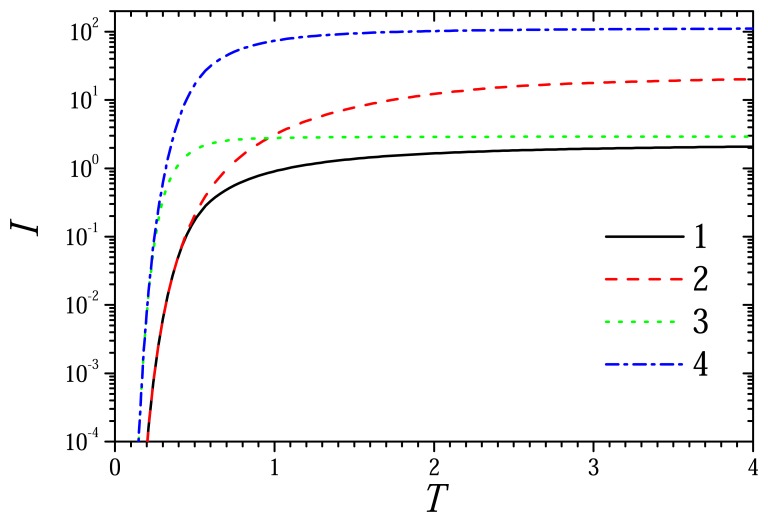
The dynamics of the dimensionless biosensor current *I*(*T*) at two concentrations of the sub-strate *S*_0_: 0.1 (1, 2), 10 (3, 4) and two concentrations of the mediator *M*_0_: 0.1 (1, 3), 10 (2, 4). Other parameters are the same as in Fig. 1.

Figs. 1 and 2 show the profiles of concentrations of the substrate, mediator and product in the enzyme membrane (*x* ∈ (0, *d_e_*), *X* ∈ (0, 1)) as well as in the external diffusion layer (*x* ∈ (*d_e_*, *d_e_* + *d_d_*), *X* ∈ (1, 1 + *δ*)) accepting *d_e_* = 100 *μ*m, *d_d_* = 300 *μ*m. The dynamics of the biosensor current is presented in Fig. 3. The biosensor action was simulated for two concentrations (0.01 and 1M) of the substrate (*s*_0_) as well as two concentrations (10^-5^ and 10^-3^ M) of the mediator (*m*_0_). The corresponding dimensionless concentrations of the substrate (*S*_0_) as well as of the mediator (*M*_0_) are: 0.1 and 10. Values of all other parameters are as defined in (36). In Figs. 1 and 2, the concentration profiles were normalized as follows:

(53) $$S_{N}=S/S_{0}=s/s_{0},\;M_{N}=M/M_{0}=m/m_{0},\;P_{N}=P/M_{0}=p/m_{0}.$$

In Figs. 1 and 2, the concentration profiles were plotted at the time *T_R_* when the process reaches steady state and the time *T*_0.5_ when 50% of the steady state current has been reached. At values (36) of the parameters, the time *t* in seconds is converted to the dimensionless time *T* by *T* = 0.03*t*.

As one can see in Fig. 1, there is a quit long shoulder in the profile of the mediator concentration (curve 5) at 1.3 < *X* < 2.3. The shoulder appears in the case of relatively high concentration *M*_0_ of the mediator and low concentration *S*_0_ of the substrate. At those conditions (*M*_0_ ≫ *S*_0_, Σ ≪ 1), the rate of the enzymatic reaction depends practically only on the substrate concentration as defined in (33). In the beginning of the biosensor action, there is no substrate in the enzyme membrane and the mediator diffuses fast from the electrode surface along the enzyme membrane and even to the bulk solution. The enzymatic reaction starts only when some substrate touches the enzyme. Due to relatively high concentration of the mediator, the reaction progresses rapidly and the concentration of the mediator inside the enzyme near the border reduces also rapidly. Consequently, for a short time the mediator concentration inside the enzyme becomes slightly lower than outside the membrane. No similar effect can be noticed in Fig. 2 which shows concentration profiles for the opposite case of (*S*_0_ ≫ *M*_0_, Σ ≫ 1). Additional numerical experiments approved that a shoulder in the profile of the mediator concentration appears only in the cases when Σ ≪ 1.

Fig. 3 shows the dynamics of the current calculated at different concentrations of the substrate and mediator. Particularly, curve 2 shows the dynamics of the response at *S*_0_ = 0.1 and *M*_0_ = 10, at which Fig. 1 shows the profiles of the concentrations of the species. The profiles of the concentrations depicted in Fig. 2 correspond to curve 3 in Fig. 3.

One can see in Fig. 3 that the biosensor current is affected by both concentrations: *S*_0_ and *M*_0_. The current grows notably faster at higher concentration *S*_0_ (curves 3 and 4) of the substrate rather than at lower one (curves 1 and 2). The effect of concentration *M*_0_ of the mediator on the biosensor response becomes notable with some delay. The mediator diffuses from the CME into the enzyme layer in a sufficient for reaction amount very quickly while the substrate has to diffuse across the Nernst diffusion and enzyme layers. Therefore, at the very beginning of the biosensor operation, the biosensor acts under a limitation of the substrate diffusion.

### 6.2. The Impact of the Diffusion Modulus

**Figure 4.**
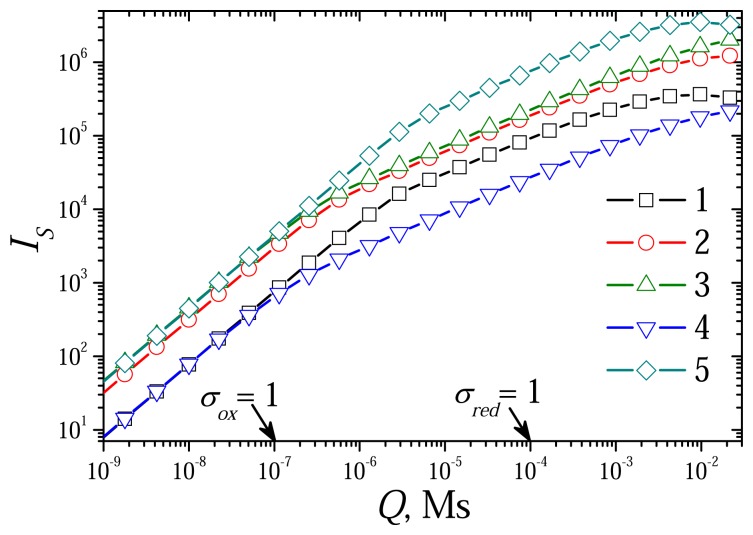
The steady state dimensionless current *I_S_* versus the reduced diffusion modulus *Q* at different concentrations of the substrate and mediator, *S*_0_: 0.1 (1), 1 (2, 4, 5), 10 (3), *M*_0_: 0.1 (4), 1 (1-3), 10 (5). Other parameters are the same as in Fig. 1.

**Figure 5.**
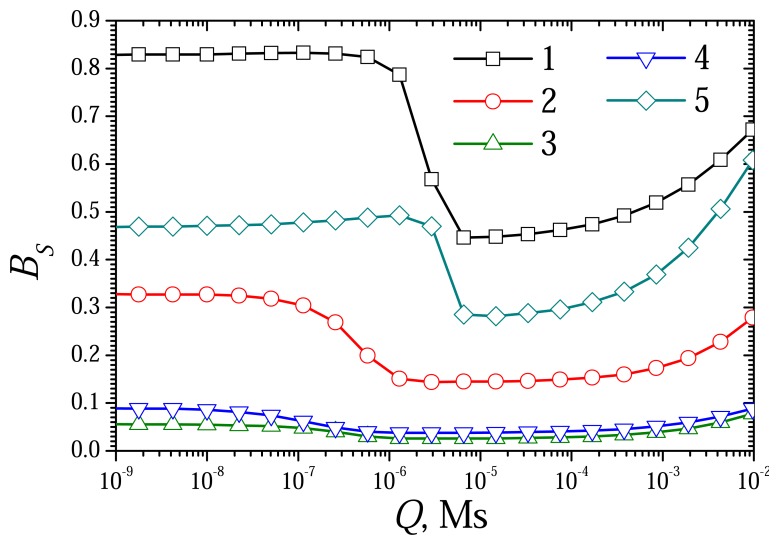
The biosensor sensitivity *B_S_* versus the reduced diffusion modulus *Q*. The parameters and notation are the same as in Fig. 4.

The dimensionless model (42)-(52) contains two diffusion modulus: *σ_red_* and *σ_ox_*. The reduced dif-fusion modulus *Q* is a common part of *σ_red_* and *σ_ox_* (see (34) and (39)). At constant rates *k_red_* and *k_ox_* of the reactions (1) and (2), it is reasonable to use the reduced diffusion modulus *Q* instead of two mod-ulus: *σ_red_* and *σ_ox_*. To investigate the effect of the diffusion modulus *Q* on the biosensor response, the biosensor action was simulated at different concentrations of the substrate and the mediator changing the enzyme layer thickness. Fig. 4 shows the dependence of the steady state dimensionless current *I_S_* on the modulus *Q*, while Fig. 5 shows the corresponding dependence of the sensitivity BS. The dependence of the response time *T*_0.5_ on *Q* is depicted in Fig. 7. The calculation was performed at three concentrations of the substrate (*S*_0_) and three concentrations of the mediator (*M*_0_) changing exponentially the thickness de of the enzyme layer from 0.3 *μ*m up to 1.5 mm. Values of all other parameters were assumed constant as defined in (36). Let us notice that accepting these values of the parameters, *σ_ox_* becomes equal to unity when *Q* = 10^–7^ Ms, and *σ_red_* = 1 at *Q* = 10^–4^ Ms.

**Figure 6.**
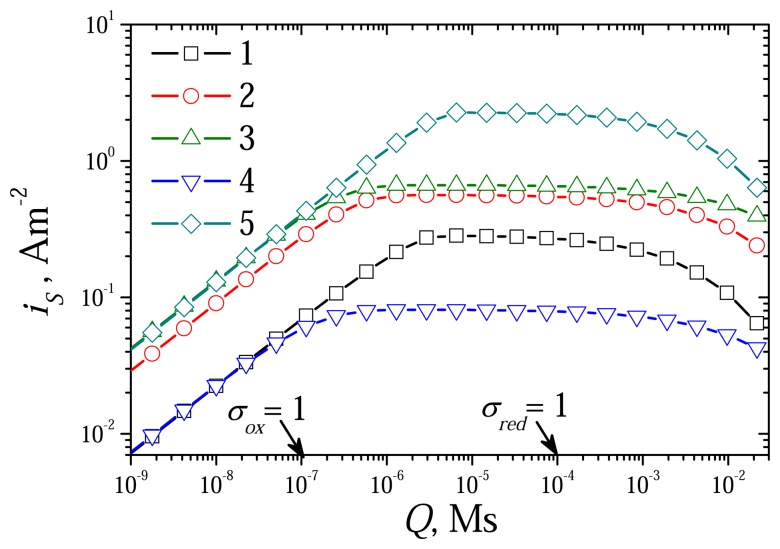
The steady state biosensor current *i_S_* versus the reduced diffusion modulus *Q*. All the parameters and notation are the same as in Fig. 4.

As one can see in Fig. 4, at small values of the diffusion modulus, *σ_ox_* < 1, the dimensionless current *I_S_* is approximately a linearly increasing function of *Q* as well as of $d_{e}^{2}$. At large values of *Q*, *σ_red_* ≫ 1, *I_S_* becomes a non-monotonous function of *Q* (curves 1 and 5). To see the behaviour of the biosensor response versus the diffusion modulus the results of calculations were re-plotted in Fig. 6 in terms of dimensional steady state current *i_S_*. Fig. 6 shows clearly the non-monotony of the steady state current *i_S_* versus the modulus *Q*. As one can see in Fig. 6, increasing *Q* from 1/*k_ox_* (*σ_ox_* = 1) up to 1/*k_red_* (*σ_red_* = 1), the steady state current *i_S_* changes slightly only. At greater values of *Q*, *σ_red_* > 1, the steady state current *i_S_* monotonously decreases.

The complex effect of the diffusion modulus on the biosensor response can be seen also in Fig. 5. In the cases when CME acts under limitation of the enzyme-mediator interaction (*σ_ox_* < 1, *Q* < 10^–7^ Ms), the biosensor sensitivity *B_S_* practically does not depend on the diffusion modulus. It means that at these conditions the biosensor sensitivity is very resistant to changes in the thickness *d_e_* of the enzyme layer as well as in the total concentration *e_t_* of the enzyme. This resistant notably decreases at higher values of the diffusion modulus. The sensitivity *B_S_* changes even non-monotonously when *Q* increases from 10^−7^ to 10^−4^ Ms, i.e. when *σ_ox_* > 1 and *σ_red_*< 1. In the cases when CME acts under control of the mass transport (*σ_red_*> 1, *Q* > 10^–4^ Ms), the biosensor sensitivity slightly increases with increase in *Q*. The diffusion modulus especially affects the sensitivity in the cases of low substrate concentration (curve 1) and of high concentration of the mediator (curve 5). This can also be observed in Fig. 6.

**Figure 7.**
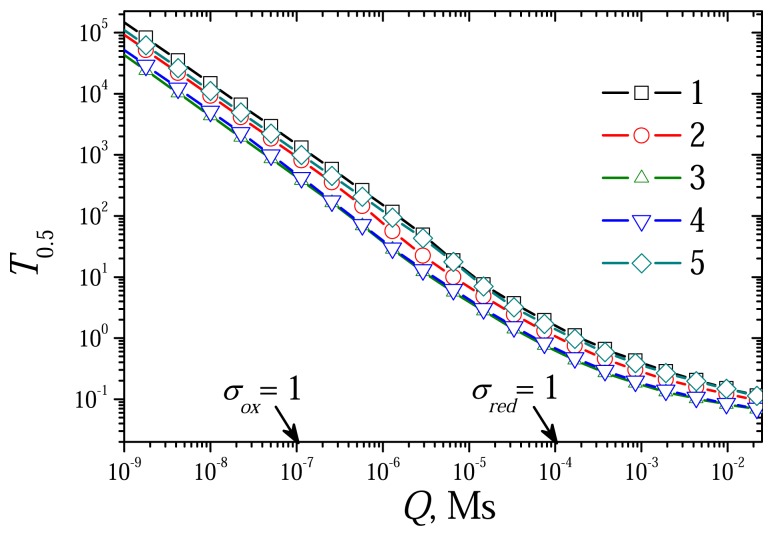
The dimensionless half-time *T*_0.5_ versus the reduced diffusion modulus *Q*. All the parameters and notation are the same as in Fig. 4.

Figs. 4, 5 and 6 show a linearity of the biosensor response in the cases when CME acts under limitation of the enzyme-mediator interaction (*σ_ox_*< 1, *Q* < 10^–7^ Ms). This linearity can also be noticed in Fig. 7. The dimensionless half-time *T*_0.5_ is distinctly a linear function of the diffusion modulus when *σ_ox_* < 1. At greater values of the diffusion modulus, the half-time *T*_0.5_ changes non-linearly.

### 6.3. The Impact of the Thickness of the External Diffusion Layer

**Figure 8.**
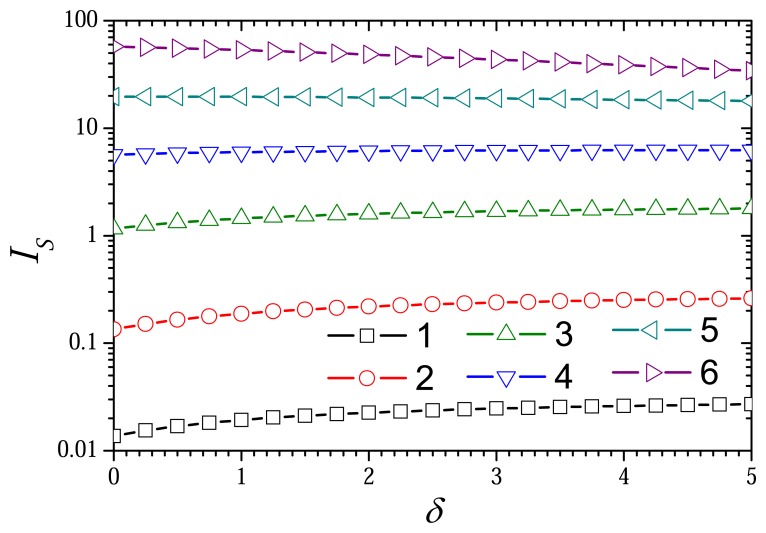
The steady state dimensionless current *I_S_* versus the dimensionless thickness *δ* of the diffusion layer at different values of the diffusion modulus $\sigma_{\text{red}}^{2}$: 10^–4^ (1), 10^–3^ (2), 10^–2^ (3), 0.1 (4), 1 (5), 10 (6). $\sigma_{\text{ox}}^{2}=10^{3}\sigma_{\text{red}}^{2}$, other parameters are the same as in Fig. 1.

**Figure 9.**
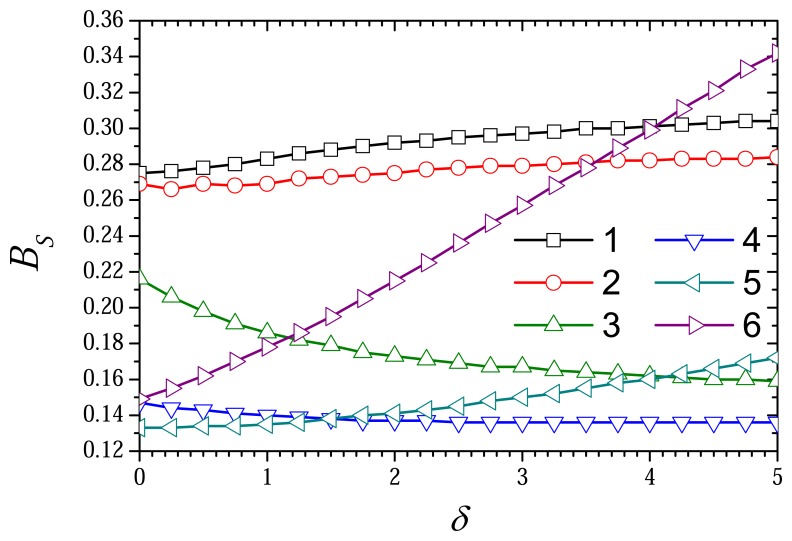
The biosensor sensitivity *B_S_* versus the dimensionless thickness *δ* of the diffusion layer at different values of the diffusion modulus. The parameters and notation are the same as in Fig. 8.

The dependence of the biosensor response on the thickness of the external diffusion layer is shown in the Figs. 8 and 9. The responses were calculated changing the thickness *d_d_* of the diffusion layer at the following six values of the thickness *d_d_* of the enzyme layer: 1, 3.16, 10, 31.6, 100, 316 *μ*m. At these values of *d_e_* keeping other parameters unchanged, the diffusion modulus ( $\sigma_{\text{ox}}^{2}$ and $\sigma_{\text{red}}^{2}$) changes in five orders of magnitude. Thus, the behaviour of the biosensor response was investigated at different limitations of the response.

As one can see in Fig. 8, the steady state dimensionless current *I_S_* is a monotonously increasing function of the dimensionless thickness *δ* of the external diffusion layer at values of the diffusion modulus of $\sigma_{\text{red}}^{2}<1$ (curves 1-4). At $\sigma_{\text{red}}^{2}>1$ (curve 6) the steady state current *I_S_* monotonously increases with increase in the thickness *δ*. In the case of $\sigma_{\text{red}}^{2}=1$, the current *I_S_* varies less than 10% when the thickness *δ* changes from 0 up to 5, and *I_S_* is even slightly non-monotonous function of *δ*. Consequently, the ratio of the enzyme-substrate interaction rate to the diffusivity is a determinant factor for the behaviour of the biosensor current as a function of the thickness of the diffusion layer. The rate of the enzyme interaction with the mediator is significantly less important than the enzyme interaction with the substrate. An increase in the thickness *δ* increases the distance between the electrode (*X* = 0) and the region (*X* = 1 + *δ*) where the concentration of the substrate is maintained constant. A change in δ makes no effect to the distance between the electrode (*X* = 0) and the region (*X* = 0) were the concentration of the mediator is maintained constant. At a concrete concentration of the mediator the dependence of the biosensor current on the thickness of the external diffusion layer is very similar to that of biosensors with no electrode modification [24, 27, 40–42].

Fig. 9 shows the dependence of the biosensor sensitivity *B_S_* on the thickness *δ* of the outer diffusion layer. When comparing Figs. 8 and 9, one can see that the effect of the thickness *δ* on the sensitivity notably differs from that on the current. In the cases when $\sigma_{\text{ox}}^{2}<1\;(\sigma_{\text{red}}^{2}<10^{-3}$, curves 1 and 2) the sensitivity increases with increase in the thickness *δ*. At higher values of the diffusion modulus when $\sigma_{\text{ox}}^{2}>1$ and $\sigma_{\text{red}}^{2}<1$ (curves 3 and 4) the sensitivity *B_S_* is an monotonously decreasing function of the thickness *δ*. In the cases when $\sigma_{\text{red}}^{2}\geq 1\;(\sigma_{\text{ox}}^{2}\geq 10^{3}$, curves 5 and 6) the sensitivity *B_S_* again becomes a monotonously increasing function of the thickness *δ*. At $\sigma_{\text{red}}^{2}=10\;(\sigma_{\text{ox}}^{2}=10^{4}$, curves 6) the sensitivity *B_S_* is especially growth function of *δ*.

A relatively short linear range of the calibration curve is one of serious drawbacks restricting wider use of biosensors [1, 2, 4]. An opportunity to increase the biosensor sensitivity as well as the linear range of the calibration curve by increasing the thickness of the external diffusion layer is an important feature of biosensor based on CME, especially due to the possibility to increase the sensitivity at different limitations of the biosensor action.

### 6.4. The Impact of the Outer Substrate Concentration

**Figure 10.**
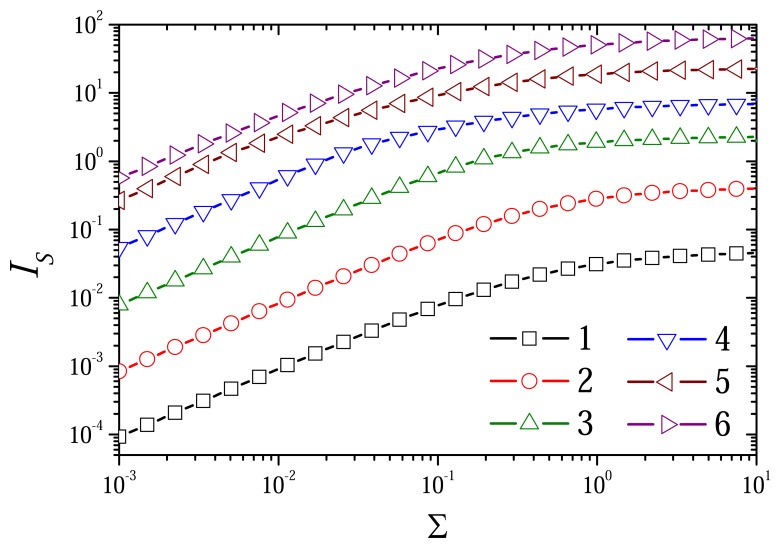
The steady state dimensionless current *I_S_* versus the ratio Σ of the substrate and mediator concentrations at different values of the diffusion modulus $\sigma_{\text{red}}^{2}$: 10^−4^(1), 10^−3^(2), 10^−2^(3), 0.1 (4), 1 (5), 10 (5), keeping constant concentration *M*_0_ = 1 of the mediator. $\sigma_{\text{ox}}^{2}=10^{3}\;\sigma_{\text{red}}^{2}$, other parameters are the same as in Fig. 1

**Figure 11.**
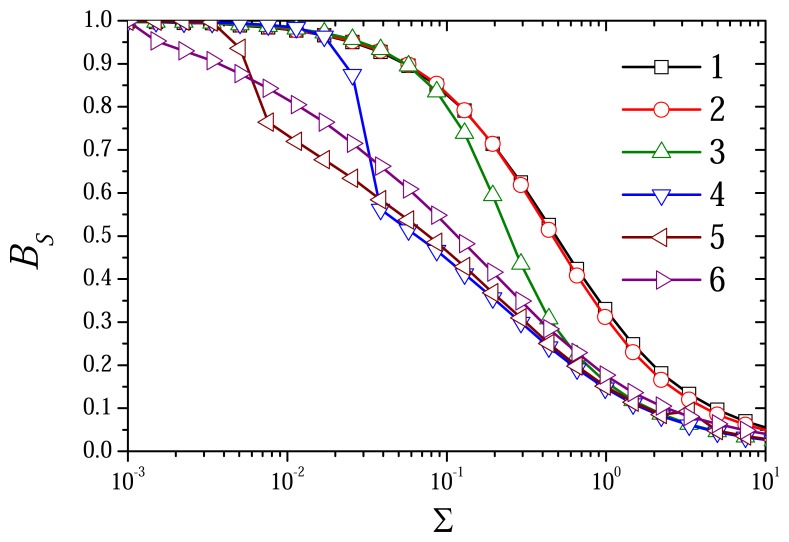
The biosensor sensitivity BS versus the ratio Σ of the substrate and mediator concentrations at different values of the diffusion modulus. The parameters and notation are the same as in 10.

**Figure 12.**
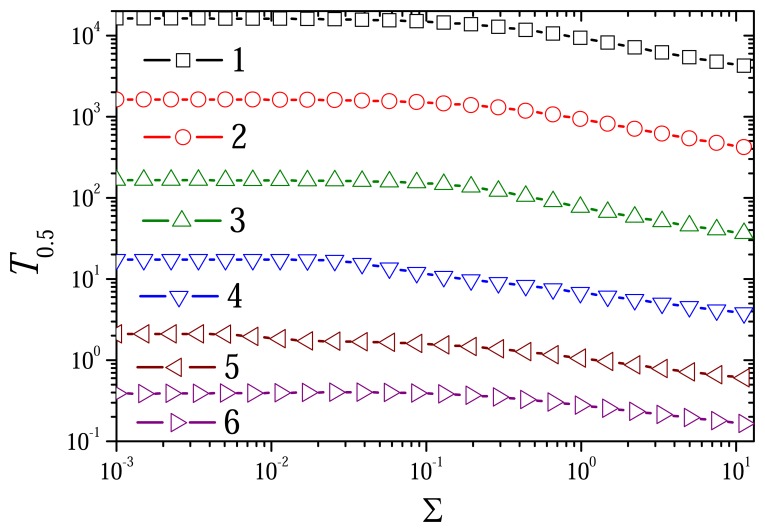
The dimensionless half-time *T*_0.5_ versus the ratio Σ of the substrate and mediator concentra-tions at different values of the diffusion modulus. The parameters and notation are the same as in Fig. 10.

The dependence of the biosensor response on the dimensionless ratio Σ of the substrate and mediator concentrations is depicted in Figs. 10, 11 and 12. The biosensor responses were simulated at different values of the diffusion modulus by changing the substrate concentration *s*_0_ in the bulk solution and keeping the mediator concentration *m*_0_ constant.

One can see in Fig. 10, a linear range of the calibration curve up to Σ ≈ 0.1 (*S*_0_ ≈ 0.1, *s*_0_ ≈ 10 mM). The dependence of the steady state current on the ratio Σ is noticeably affected by the diffusion modulus. The current is directly proportional to $\sigma_{\text{red}}^{2}$ as well as to $\sigma_{\text{ox}}^{2}$. At low values of the diffusion modulus, tenfold increase in $\sigma_{\text{red}}^{2}$ increases the steady state dimensionless current *I_S_* approximately also tenfold (curves 1-3). However, at $\sigma_{\text{red}}^{2}\geq 1$ (curves 4-6), the effect of the diffusion modules on the steady state current *I_S_* notably decreases. When $\sigma_{\text{red}}^{2}$ increases from 10 (curve 5) to 100 (curve 6), the current *I_S_* increases only about 2-3 times. This is also can be noticed in Fig. 7.

Fig. 11 shows that the biosensor sensitivity notably decreases with an increase in the ratio Σ of the substrate and mediator concentrations at all values of the diffusion modulus. In general, the effect of the sensitivity decrease increasing the substrate concentration is rather well known [1, 2]. As usually, the biosensors are highly sensitive at very low concentrations of the substrate (Σ ≤ 10^–2^) and they are of very low sensitivity a high concentrations of the substrate (Σ > 1). This effect can also be noticed in Fig. 10.

One can see no notable difference between the shapes of curves 1 and 2 in Figs. 10 and 11. So, in the cases when $\sigma_{\text{ox}}^{2}\leq 1\;(\sigma_{\text{red}}^{2}\leq 10^{-3}$, curves 1 and 2) the diffusion modulus practically has no influence on the biosensor sensitivity. When $\sigma_{\text{ox}}^{2}>1$ and $\sigma_{\text{red}}^{2}\leq 1$ (curves 3-5) the sensitivity *B_S_* decreases with increase in the diffusion modulus. The diffusion modulus especially effects the biosensor sensitivity at moderate concentrations of the substrate (0.01 ≤ Σ ≤ 1). When the response is under diffusion control ( $\sigma_{\text{red}}^{2}>1$, curve 6) the sensitivity slightly increases. This was very shown in Fig. 5.

Fig. 12 shows the effect of the ratio Σ of the substrate and mediator concentrations on the dimension-less half-time *T*_0.5_. No notable effect is observed at low values of Σ. The value of Σ at which *T*_0.5_ starts to decrease depends on the diffusion modulus. A constant range of *T*_0.5_ increases with an increase in the diffusion modulus.

### 7. Conclusions

The mathematical model (3)-(26) of an amperometric biosensor based on a chemically modified elec-trode can be successfully used to investigate the kinetic peculiarities of the biosensor response. The corresponding dimensionless mathematical model (42)-(52) can be used as a framework for numerical investigation of the impact of model parameters on the biosensor action and to optimize the biosensor configuration.

The biosensor current grows notable faster at higher substrate concentrations in the bulk solution than at lower ones (Fig. 3). At the very beginning of the operation, the biosensor acts under a limitation of the substrate diffusion from the bulk solution to the electrode.

A value of the diffusion modulus substantially determines the behaviour of the response and sensi-tivity of the biosensor. The steady state biosensor current is a nonsmonotonous function of the diffusion modulus (Fig.6). In the cases when $\sigma_{ox}^{2}\leq 1$, the diffusion modulus practically has no influence on the biosensor sensitivity. When $\sigma_{ox}^{2}>1$and $\sigma_{\text{red}}^{2}\leq 1$, the sensitivity changes non-monotonously with the diffusion modulus. When the response is fully under diffusion control $\left(\sigma_{\text{red}}^{2}>1\right)$, the sensitivity slightly increases with increase in the diffusion modulus (Fig. 5 and 11).

The ratio of the enzyme-substrate reaction rate to the diffusion rate (the diffusion modulus $\sigma_{\text{red}}^{2}$) is the determinant factor for the behaviour of the biosensor current as a function of the dimensionless thickness of the external diffusion layer. At $\sigma_{\text{red}}^{2}<1$ the steady state dimensionless current *I_S_* is a monotonously increasing function of the dimensionless thickness *δ* of the external diffusion layer. At $\sigma_{\text{red}}^{2}>1$ the current *I_S_* decreases with increase in the thickness *δ*. In the case of $\sigma_{\text{red}}^{2}=1$, the current *I_S_* varies slightly and in a non-monotonous way (Fig. 8). The biosensor sensitivity as well as the linear range of the calibration curve can be increased by increasing the thickness of the external diffusion layer (Fig. 9).
